# COVID‐19, varying genetic resistance to viral disease and immune tolerance checkpoints

**DOI:** 10.1111/imcb.12419

**Published:** 2020-11-23

**Authors:** Christopher C Goodnow

**Affiliations:** ^1^ Garvan Institute of Medical Research Sydney NSW Australia; ^2^ Cellular Genomics Futures Institute UNSW Sydney Sydney NSW Australia

**Keywords:** cGAS, CTLA‐4, interferon, PD‐1, pyroptosis, TNFAIP3

## Abstract

Coronavirus disease 2019 (COVID‐19) is a zoonosis like most of the great plagues sculpting human history, from smallpox to pandemic influenza and human immunodeficiency virus. When viruses jump into a new species the outcome of infection ranges from asymptomatic to lethal, historically ascribed to “genetic resistance to viral disease.” People have exploited these differences for good and bad, for developing vaccines from cowpox and horsepox virus, controlling rabbit plagues with myxoma virus and introducing smallpox during colonization of America and Australia. Differences in resistance to viral disease are at the core of the severe acute respiratory syndrome coronavirus 2 (SARS‐CoV‐2) crisis, yet our understanding of the mechanisms in any interspecies leap falls short of the mark. Here I review how the two key parameters of viral disease are countered by fundamentally different genetic mechanisms for resistance: (1) virus transmission, countered primarily by activation of innate and adaptive immune responses; and (2) pathology, countered primarily by tolerance checkpoints to limit innate and adaptive immune responses. I discuss tolerance thresholds and the role of CD8 T cells to limit pathological immune responses, the problems posed by tolerant superspreaders and the signature coronavirus evasion strategy of eliciting only short‐lived neutralizing antibody responses. Pinpointing and targeting the mechanisms responsible for varying pathology and short‐lived antibody were beyond reach in previous zoonoses, but this time we are armed with genomic technologies and more knowledge of immune checkpoint genes. These known unknowns must now be tackled to solve the current COVID‐19 crisis and the inevitable zoonoses to follow.

## Graphical abstract

Coronavirus disease 2019 (COVID‐19) results from a virus making an interspecies leap that tests genetic resistance to viral disease, like most of the great plagues sculpting human history. Here I set out known unknowns for tackling COVID‐19, taking advantage of progress understanding how viral disease is countered by fundamentally different genetic mechanisms for resistance: resistance to virus transmission primarily by activation of immune responses; and resistance to pathology primarily by tolerance checkpoints to limit immune responses.

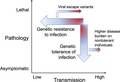

## Introduction

“It is likely that the precursor of SARS‐CoV has repeatedly crossed the species barrier but only occasionally has it succeeded in adapting to human–human transmission. This adaptation clearly occurred in late 2002 and it may happen again in the future. But given the present understanding and awareness about SARS, we expect that such re‐emergence is unlikely to lead to a global outbreak on the scale of 2003.”^1^

The 2003 severe acute respiratory syndrome (SARS) outbreak infected a cumulative total of 8098 people worldwide, of whom 774 died. As history now shows, our understanding of SARS and the cause and treatment of zoonotic coronavirus disease falls short of the mark. Coronavirus disease 2019 (COVID‐19) is a new human disease closely related to the SARS pandemic of 2003. Like SARS, COVID‐19 is caused by a zoonotic jump of a bat coronavirus to humans followed by adaptation for high human‐to‐human transmission, yielding a new virus called severe acute respiratory syndrome coronavirus 2 (SARS‐CoV‐2). As of 17 November 2020, SARS‐CoV‐2 has infected more than 55 million people globally, of whom more than 1.3 million people have died.

## Varying SARS‐CoV‐2 pathology is the root of the COVID crisis

Public health responses quickly countered and eradicated the two successive zoonotic outbreaks of SARS in 2003 and 2004 because there were very few asymptomatic virus shedders. The great majority of SARS‐infected people required medical attention, including 20–30% requiring intensive care, and symptoms and fever almost always preceded onset of virus shedding.^1^ This made it possible to stop virus transmission by identifying infected individuals quickly, isolating them and quarantining their close contacts.

SARS‐CoV‐2 has proved much harder to contain because of a wider spread in clinical presentation: 80% of people have mild or asymptomatic infections, but an unmanageably high percentage develop severe disease requiring hospitalization including 5–10% requiring intensive care. Severe COVID‐19 disease is broad based and unpredictable. While age is a risk factor as it was for SARS (discussed later), 30% of hospitalized cases in New South Wales at the end of March 2020 were people under 60 years. In the Chinese analysis of 72 314 cases “14.8% of confirmed cases among health workers were classified as severe or critical.”^2^ These high hospitalization rates are coupled with early, presymptomatic virus shedding,^3^ high transmissibility and a high frequency of asymptomatic virus shedders measured at between 18% and 33%.^4^, ^5^, ^6^, ^7^, ^8^

In Australia, these unique epidemiological parameters have forced Federal and State governments to deviate from well‐prepared influenza pandemic response plans.^9^ In the 2009 H1N1 influenza “Swine Flu” pandemic, hospitalization rates were 40 times lower than for infection with SARS‐CoV‐2.^10^ The Australian pandemic plan assumed “As clinical severity increases the following will also increase: the proportion of infected individuals seeking treatment, which means the public health interventions to reduce ongoing transmission that rely on identification of cases will likely be more effective.” The curve ball thrown by SARS‐CoV‐2 is that this assumption, which did apply for SARS, does not apply for the current pandemic: a high proportion of virus‐shedding individuals have mild or no illness, do not seek treatment and cannot be identified short of highly efficient contact tracing or population‐wide virus testing.^11^

## Interspecies leaps reveal imbalances in host genetic resistance to viral disease

What explains the range of clinical outcomes when viruses leap into a new species, from asymptomatic to lethal? Historically it has been ascribed to differing levels of “genetic resistance to viral disease” in the new host. To clarify, in this review the word “genetic” is used in the sense of “encoded in the genome,” even if the relevant gene and trait is not known to be variable within a species. The nature of resistance is a major gap in the knowledge needed to reduce the rates of intensive care unit admissions for COVID‐19 and to promote durable immunity to eradicate the virus with a vaccine.

**Figure 1:**
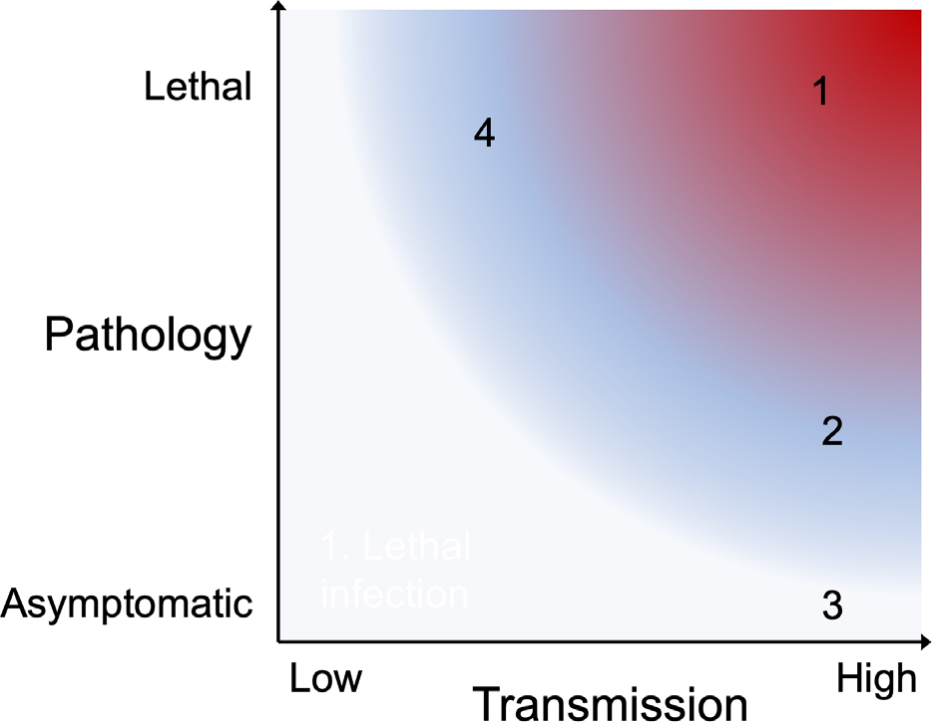
Key zoonotic disease parameters: varying transmission between individuals, and varying pathology among infected individuals. (1) Highly transmissible epidemics with high morbidity and mortality, exemplified by SARS‐CoV‐2, SARS and smallpox. (2) Highly transmissible but low morbidity and rarely lethal infections, exemplified by the 2009 H1N1 influenza epidemic, common cold coronaviruses, Epstein‐Barr virus, cowpox and horsepox. (3) Highly transmissible but asymptomatic infections, exemplified by SARS‐related coronaviruses in bats, and by commensal viruses and bacteria. (4) Lower transmission but highly morbid or lethal infections, exemplified by human immunodeficiency virus and anthrax. SARS‐CoV‐2, severe acute respiratory syndrome coronavirus 2.

It is valuable to split apart virus transmission and host pathology as separate parameters of infectious disease (Figure 1) because, as described later, they are countered by host genetic resistance mechanisms that are fundamentally different in nature and in stability over time. SARS‐CoV‐2 and SARS are devastating when they leap into the human population because they are highly transmissible between individuals and cause morbid or lethal disease in a high percentage of infected individuals. Many previous, history‐shaping zoonoses fall in this category, exemplified by smallpox. However, the precursors of these viruses before their zoonotic leap, exemplified by the bat coronavirus precursors of SARS and SARS‐CoV‐2 or by cowpox virus, display high transmission but low morbidity or mortality. Thus, disease appears to have more to do with the host than the virus itself.

## Genetic resistance to virus transmission

**Figure 2:**
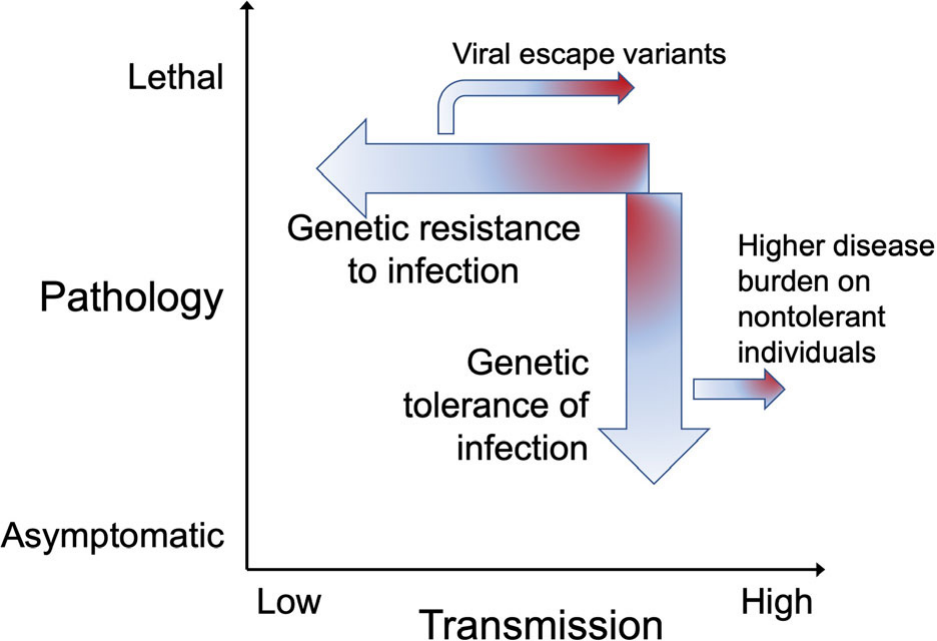
Mechanisms for genetically encoded resistance to communicable disease: (1) resistance to virus transmission and (2) tolerance to viral infection by diminishing pathological responses.

A range of genetically encoded mechanisms counter transmission of a new virus within the population (Figure 2), including absence of virus receptors on host cells, innate immune responses and adaptive immune responses, notably neutralizing antibodies.

Genetic resistance conferred by absence of virus receptors on host cells is best illustrated by “elite resistors” of human immunodeficiency virus infection who inherit two alleles of a *CCR5* gene deletion. Significantly, the prevalence of this *CCR5* allele in Europeans may have been driven by conferring lower mortality to smallpox.^12^ It is not known whether loss of CCR5 deprives variola virus of a coreceptor or diminishes an otherwise lethal immune response to the virus through its physiological role in transmitting inflammatory chemokine signals. A key step in the leap of coronaviruses from bats to humans was acquisition of mutations in the virus spike protein to allow it to bind more avidly to the human version of the host cell receptors, ACE2 for SARS‐CoV‐2 and SARS‐CoV or DPP4 for Middle East respiratory syndrome‐related coronavirus (MERS‐CoV).^13^ There is no information yet about whether some people may resist SARS‐CoV‐2 infection because of differences in the *ACE2* receptor gene.

The central role of adaptive antibody responses in blocking virus transmission is highlighted by Sabin’s attenuated oral poliovirus strains, which have been given to millions of children to eradicate polio from most countries around the world. These cause an asymptomatic infection and transient virus shedding from the gut, while triggering durable neutralizing antibodies that block infection and transmission of wild poliovirus. However, in people with genetic deficiencies in antibody formation, such as X‐linked agammaglobulinemia, the attenuated virus causes a persistent asymptomatic infection with intestinal virus shedding for up to 18 years.^14^, ^15^ Control of measles virus transmission hinges upon the remarkable ability of most people to maintain neutralizing antibody titers above 1/1000 for decades, and those titers are a key quality control for intravenous gamma globulin given to people with genetic deficiencies in antibody formation.^16^, ^17^

Genetic resistance to communicable disease by inhibiting microbe transmission, whether by loss of receptors or by gaining immune responses, is nevertheless unstable over time because variant microbes are rapidly selected that evade resistance and transmit better, as plant breeders have known for more than half a century.^18^, ^19^ We are each reminded annually of the unstable state of resistance based on blocking virus transmission. It is the reason we need a different seasonal influenza vaccine each year. Neutralizing antibodies are strongly and durably elicited by influenza, driving strong selection for virus mutations and “antigenic drift” in the virus epitopes so that they are no longer recognized by our current set of antibodies.

Coronaviruses have the largest genomes among RNA viruses and, unlike influenza virus or retroviruses, replicate their genome with much higher fidelity.^20^ SARS‐CoV‐2 acquires point mutations at a rate of approximately 1 per 10 000 bases per year.^21^ Consequently, coronaviruses do not appear to exploit antigenic drift as an efficient mechanism to escape neutralizing antibody formation in the way that so drastically limits acquired immunity and vaccines against influenza or human immunodeficiency virus. Instead, production of neutralizing antibodies is relatively low and curiously short‐lived after human infection with the common cold coronavirus HCoV‐229E^22^, ^23^ or after clinically severe infections with SARS‐CoV.^24^, ^25^ Short‐lived antibody formation also limits the utility of live attenuated vaccines against infectious bronchitis coronavirus in the poultry industry.^26^, ^27^ Whereas influenza escapes our mechanisms for neutralizing antibody production by high viral genome mutation rates, coronaviruses avoid the fitness cost of high mutation rates by somehow manipulating our mechanisms for neutralizing antibody responses so that they are low titer, short lived and do not stop virus transmission in the population.^23^

## Genetic resistance to virus‐induced pathology

Instead of blocking transmission, an alternative and more evolutionarily stable strategy involves genetic mechanisms to diminish pathological host responses to infection (Figure 2), referred to as “pathogen tolerance” by plant breeders.^18^, ^19^ It involves homeostatic mechanisms—immune tolerance checkpoints—to raise thresholds for initiating and continuing innate or adaptive immune responses, to physically separate replicating microbes from immune response receptors in host cells and to repair damaged barriers and tissues. High genetic tolerance to SARS coronavirus infections may explain why bats asymptomatically transmit these viruses at high rates, as detailed later.

Disease resistance by tolerating microbe replication is evolutionarily more stable because it can be achieved without decreasing microbe transmission and therefore without selecting for resistance escape variants.^18^, ^19^ Tolerance mechanisms are also more stable because they are driven to become genetically fixed in the host species. As more individuals tolerate but transmit the virus (asymptomatic shedders) it increases infection rates in the remaining individuals who are genetically unable to tolerate infection. These individuals die out, leaving only tolerant hosts. An example of this form of “germ warfare” is displacement of ladybeetle species by invasive species asymptomatically carrying and transmitting a high burden of fungal pathogens as a result of genetically heightened innate immune responses.^28^ Typhoid Mary is a famous example of an individual with low resistance to microbe transmission but high tolerance to the disease: an asymptomatic, lifelong superspreader of *Salmonella enterica* causing many severe or lethal cases of typhoid.^29^

Contrary to popular view, CD8 T lymphocyte responses to viruses may primarily represent a genetic mechanism to tolerate infection. Unlike neutralizing antibodies to influenza, the strong CD8 T‐cell responses to conserved influenza epitopes do not appear to diminish virus transmission as evidenced by the need to update the seasonal influenza vaccine annually. However, these T cells may account for the diminished morbidity after a new influenza zoonosis becomes established in the population. Transmission of ectromelia virus in mice, a close relative of variola virus (the agent of smallpox, discussed later), is primarily blocked by neutralizing antibody but not by CD8 T cells: however, in the absence of CD8 T cells infected mice do not tolerate infection and rapidly succumb to lethal inflammatory disease.^30^, ^31^

Epstein‐Barr virus is widely transmitted and establishes a lifelong infection in most people, which we nevertheless tolerate asymptomatically by maintaining very high numbers of circulating CD8 T cells against the virus. These CD8 T‐cell responses do not develop in people who inherit a genetic defect in *SH2D1A*, which encodes SLAM‐associated protein, an intracellular adaptor for SLAM‐family receptors on CD8 T cells.^32^ In *SH2D1A*‐deficient people, Epstein‐Barr virus provokes morbid pathological immune responses resulting in severe mononucleosis and, in some, a lethal “cytokine storm” terminating in the macrophage hyperactivation syndrome of hemophagocytic lymphohistiocytosis.^33^, ^34^, ^35^

The importance of considering resistance to transmission and resistance to viral pathology separately is as important for SARS‐CoV‐2 as it was for polio. The first‐generation inactivated Salk vaccine did not protect against poliovirus infection and fecal–oral transmission but did confer resistance to viral infection of the central nervous system and paralyzing poliomyelitis.^36^, ^37^ This property of suppressing index clinical cases but not virus transmission has led to silent outbreaks of wild poliovirus.^38^ By contrast, the second‐generation Sabin oral poliovirus vaccine blocks transmission and became the cornerstone of poliovirus control, but cannot be used alone in the endgame of virus eradication because of rare reversion to a paralysis‐inducing strain.^36^

In experimental challenge studies in nonhuman primates, a nonreplicating adenovirus vector encoding the SARS‐CoV‐2 spike elicited virus‐reactive CD8 T cells but only low (less than 1/100) titers of neutralizing antibodies, which were insufficient to protect any of the animals from virus infection in the upper respiratory tract but diminished virus replication and pathology in the lung.^39^ A similar MERS vaccine failed to protect any camels from natural transmission of MERS coronavirus.^40^ This raises the possibility that first‐generation SARS‐CoV‐2 vaccines, like the inactivated polio vaccine, may be a step forward by diminishing COVID‐19 hospitalization rates, but potentially a step backward by fostering “silent” outbreaks that put pressure on vulnerable unvaccinated populations and people less able to mount a CD8 T‐cell response.

## Myxomatosis: a history‐shaping example of varying genetic resistance

The paradigm‐setting example of varying genetic resistance when viruses leap between species was the deliberate release of myxoma virus in Australia to control European *Oryctolagus* rabbit populations.^41^ Myxoma virus causes a mild disease but extended virus replication localized to the site of inoculation in its natural host, South American *Sylvilagus* rabbits, but caused over 99% lethal systemic disease when introduced to *Oryctolagus* rabbits. After seven successive epizootic outbreaks of myxomatosis, the recovering Australian rabbit population was genetically more resistant to lethal disease, despite the virus replicating equally well in cells from genetically resistant or susceptible *Oryctolagus* rabbits.^42^, ^43^ Targeted exome sequencing of DNA from genetically susceptible and resistant *Oryctolagus* rabbits revealed convergent selection for chromosomal segments containing pre‐existing gene variants in immune regulators including *IFNA*, *CD96*, *FCRL3* and *MHC‐I*.^44^ Interferon alpha has both antiviral and immune‐modulatory roles, and the MHC genes control the specificity and magnitude of immune responses and immune tolerance. Interestingly, CD96 is an inhibitory receptor on natural killer cells and T cells contributing to an immune tolerance checkpoint restraining immune responses to cancer cells.^45^, ^46^ Similarly, FCRL3 is an inhibitory receptor on B cells and FOXP3^+^ regulatory T cells with common inherited variants associated with susceptibility to multiple autoimmune diseases.^47^, ^48^ These genetic correlations are nevertheless only the starting point for understanding varying host tolerance to interspecies virus transmission.

## Varying genetic resistance to smallpox, cowpox and horsepox: the birth of vaccination

Myxoma virus release was, very regrettably, the second history‐shaping exploitation of differing host genetic tolerance to viruses in Australia. The first was an unexplained release of variola virus into the indigenous Sydney aboriginal community by the first fleet of European colonists. The likely source was ship surgeons’ stocks of preserved smallpox scabs used for variolation.^49^ The release caused a smallpox epidemic in 1789 that decimated approximately 90% of the first Australians over large parts of southeast Australia,^49^ opened a path for colonization by Europeans and contributed to loss of 60 000 years of indigenous agricultural knowledge.^50^ Like earlier European experience employing variola virus as germ warfare against indigenous North Americans,^51^ morbidity and mortality were much greater in indigenous Australians than when variola virus was introduced into immunologically naïve populations of European descent. The basis for individual differences in smallpox disease severity remains unknown: is it too little or too much of an immune response?

Like COVID‐19, smallpox started as a zoonosis approximately 3000 years ago when variola virus evolved selective replication in the human population after leaping from rodents. Around the same time and location, camels were introduced to the Horn of Africa and closely related viruses evolved to replicate selectively in camels (camelpox virus) and in African naked‐sole gerbils (taterapox virus).^52^ Concentration of different species, subject to crowding and environmental stress, may also have been the conditions in the Arabian peninsula that enabled MERS‐CoV to leap from bats to camels to humans, and the conditions in wildlife markets in China that led SARS‐CoV to leap from bats to civets to humans.^13^

In contrast to the clinical severity of human variola infection, its predecessor approximately 10 000 years ago is represented today by cowpox virus,^52^ which propagates in rodents and causes mild human disease limited to a few skin pocks or pustules. Despite its mild clinical presentation, cowpox infection induces acquired resistance to smallpox, a folklore observation about milkmaids that led Jenner to invent “vaccination” by inoculating people with material from cowpox pustules.^53^

Ironically, cowpox is not the vaccine strain employed to eradicate smallpox from the globe. Vaccinia virus—the strain used for eradication—was revealed by genome sequencing to have diverged from cowpox virus approximately 8000 years ago, around the same time as variola virus, but diverged only in the last 300 years from horsepox virus—a now extinct virus that caused pocks and pustules on the legs of horses referred to as “grease heel” in the 18th century.^52^, ^54^, ^55^ The horse origins of vaccinia virus trace to Jenner’s original observation that horsepox spread via milkmaids to cows causing cowpox.^53^ This led Jenner and 19th century colleagues to interchangeably practice “equination” with horsepox pustules directly into humans and “vaccination” after propagating the horsepox in cows or calves before inoculating humans.^56^

Like cowpox and horsepox infections, almost all humans are genetically resistant to systemic disease caused by vaccinia virus infection. Inoculation causes only a mild, localized virus replication in a skin pock but nevertheless induces high titers of neutralizing antibodies that continue to be produced for many decades,^57^ conferring durable immunity to smallpox infection and transmission. It is these properties that underpinned the global eradication of smallpox^51^: an achievement that, in economic terms alone, has paid for all past and future investment in medical research.

Poxviruses, along with herpesviruses such as Epstein‐Barr virus, have the largest genomes of all DNA viruses and carry numerous genes encoding proteins that manipulate host innate and adaptive antiviral immune responses. Variola virus has lost parts of the genome present in its more benign cowpox virus ancestor.^52^ It is conceivable that these gene losses cause a failure to activate immune tolerance checkpoints, unleashing lethal systemic inflammatory responses to the virus.

## Genetic resistance to coronavirus pathology in bats

The coronavirus precursors of SARS‐CoV, SARS‐CoV‐2 and MERS‐CoV appear to be carried and shed for prolonged periods by many bats without causing disease, indicating high tolerance to coronavirus infection in these species.^58^, ^59^, ^60^ While the order Chiroptera is complex and diverse, genomics has provided clues to the apparent genetic resistance to coronavirus pathology displayed by members of the Myotinae and Pteropodidae bat suborders. A number of bat species are unique among mammals in having lost or partially inactivated genes encoding critical proteins for sensing viral nucleic acid in the cytoplasm and triggering interferon, pyroptosis and systemic inflammatory disease.^61^ These inherited differences include decay of many type 1 interferon genes and constitutive expression of *IFNA* genes,^62^ positively selected variants in *RNASEL* and *OAS* genes,^63^ independent deletions eliminating the *AIM2/PYHIN* genes,^64^ diminished *NLRP3* inflammasome expression and activity^65^ and a variety of *TMEM173* Ser358 substitutions causing partial loss of function in stimulator of interferon genes (STING; gene symbol *TMEM173*).^66^ Bat species have also lost most or all of the activating and inhibitory receptors found on the surface of natural killer cells in humans and other mammals.^61^ It nevertheless remains to be demonstrated through experiment that these genetic differences affect coronavirus transmission and pathology.

## Pathology is a cost of genetic resistance by immune responses

Genetic resistance to microbe transmission conferred by innate or adaptive immune responses imposes a fitness cost in the form of collateral damage to host tissues and extra energy demands.^18^, ^19^, ^67^, ^68^ This trade‐off leads to a “goldilocks” situation where too little or too much immune response is detrimental.

The innate immune response of pyroptosis highlights how the host cell’s response to virus or bacterial replication can be much more cytopathic than the microbe itself. A web of intracellular sensors of viruses, bacteria or host cell damage converge to activate caspase cleavage of a host cell protein, gasdermin D (GSDMD). The cleaved N‐terminal GSDMD fragment oligomerizes into plasma membrane pores causing inflammatory host cell death—pyroptosis.^69^, ^70^, ^71^, ^72^ The resulting GSDMD pores release the cytokine interleukin‐1 (IL‐1) from the infected cell’s cytoplasm, and IL‐1 spreads locally and systemically to cause fever, costimulate T cells^73^, ^74^ and attract neutrophils and macrophages to produce oxygen‐free radicals, degrading enzymes and other inflammatory cytokines such as IL‐6.^75^

Inherited variants in *GSDMD* illustrate how differences in genetic tolerance to infectious disease can arise. Phenotypic screening of mouse populations for genetic resistance to pyroptosis revealed a *Gsdmd* missense variant, I105N, that preserves GSDMD expression, cleavage and binding to membranes, but slows the rate of membrane pore assembly.^69^, ^72^ Another resistant pedigree inherited a mutation‐deleting part of interferon regulatory factor 2 (*IRF2*) resulting in low transcription of *Gsdmd* messenger RNA,^76^ revealing how genetic tolerance to activation of the interferon response^77^ also raises the threshold for activating pyroptosis. Mice inheriting complete *Gsdmd* genetic deficiency have 50‐fold greater rotavirus replication and more severe diarrhea because infected intestinal epithelial cells are unable to activate pyroptosis upon sensing viral RNA through NLRP9B.^78^ By contrast, *Gsdmd* genetic deficiency is beneficial in sepsis models where it confers resistance to lethal disease^69^ by preventing pyroptotic release of tissue factor by macrophages and thereby averting disseminated intravascular coagulation.^79^, ^80^

Apparently intrinsic traits of genetic resistance to virus replication turn out to reflect carefully regulated innate immune responses within the infected cells. Replication of myxoma and vaccinia viruses in cells from most mammalian species is aborted by “intrinsic” host cell restriction genes, *SAMD9* and *SAMD9L*, against which both viruses have evolved unique evasion proteins that bind with high affinity to the SAMD9/SAMD9L proteins and block their antiviral effect.^81^, ^82^, ^83^, ^84^ The SAMD9 proteins contain nucleic acid‐binding domains and are members of a diverse, ancient family of host cell proteins, the STAND domain proteins, which includes potent intracellular sensors of infection and activators of inflammation and cell death such as NOD2 and APAF1.^85^ Exactly how SAMD9 proteins sense and stop virus replication are still being worked out, but like pyroptosis their action is kept under tight checkpoint control. That control is broken in children born with *de novo* truncating mutations in *SAMD9L* that appear to remove negative regulatory domains, causing a severe autoinflammatory disorder^86^ (A Russell, C Goodnow, P Gray, unpublished data).

## Tolerance thresholds to limit pathological immune responses

**Figure 3:**
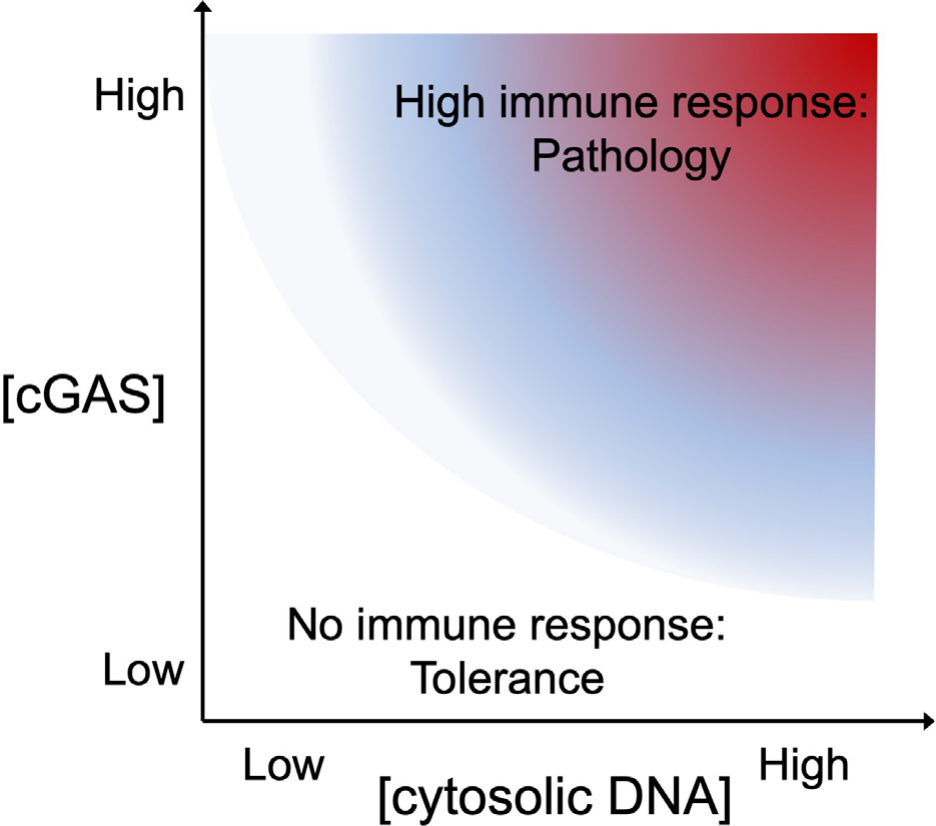
Thresholds for tolerance or immune response activation by the cytoplasmic DNA sensor protein cGAS. cGAS, cyclic GMP–AMP synthase.

The simplest demonstration of genetically tuned thresholds to tolerate virus replication comes from another intracellular virus sensing protein, cyclic GMP–AMP synthase (cGAS). cGAS binds and is activated by DNA in the cytoplasm, which could either be replicating viral DNA or short fragments of self‐DNA that have escaped from the cell’s nucleus or mitochondria.^87^ To help distinguish virus from self, a disordered DNA‐binding N‐terminal domain in cGAS remains inactive unless it binds a critical length and concentration of DNA and cGAS protein exceeds a critical concentration (Figure 3). Above these thresholds, the N‐terminal domains of individual cGAS proteins drive a phase transition into polymerized liquid droplets that dramatically increase enzymatic production of the second messenger, cyclic GMP–AMP. Cyclic GMP–AMP activates STING (gene symbol *TMEM173*), which in turn activates TAK1, IRF3, interferon and inflammatory responses. As with the aforesaid SAMD9L example, people who inherit *TMEM173* point mutations that lower the threshold for STING activation develop a severe autoinflammatory disease.^88^

Devastating mucosal inflammatory disease in people^89^ or mice^90^, ^91^, ^92^ with genetic defects in *TNFAIP3* exemplifies a disease caused not by microbes *per se* but by loss of host tolerance to these microbes. When innate or adaptive immune receptors are stimulated by microbes or self‐constituents, they initiate signaling pathways to activate the transcription factor nuclear factor kappa B, which initiates diverse immune responses but also induces *TNFAIP3* messenger RNA. TNFAIP3 protein, also called A20, serves as a potent inhibitory feedback to dampen nuclear factor kappa B activation. Without this checkpoint in intestinal epithelial cells and macrophages, commensal bacteria in the gut stimulate an overexuberant immune response through a vicious cycle that progressively thins the mucin layer and epithelial layer that normally serve as barriers physically separating commensal gut bacteria from inflammatory immune cells.^90^, ^91^

Subtle inherited *TNFAIP3* gene variants illustrate the goldilocks problem of genetically varying immune tolerance.^92^ An inherited *TNFAIP3* variant in mice that lessens the protein’s inhibitory activity decreased immune tolerance to self‐tissues and caused subclinical colitis, but also increased resistance to virus replication and otherwise lethal disease caused by Coxsackievirus B4. However, the same gene variant caused a fatally overexuberant immune response when the mice were infected with ectromelia virus. The right amount of immune response to one virus is too much for another.

An ancient human *TNFAIP3* variant from extinct Denisovan hominins similarly decreases the activity of the TNFAIP3 checkpoint.^92^ This Denisovan allele is common in modern people throughout Oceania including the first Australians, and displays the genetic signatures of beneficial selection after modern humans migrated across the Wallace line to live among Australian fauna and flora 60 000 years ago. Perhaps people encountered new zoonotic viruses from the Australian fauna, or alternatively by escaping from pathogenic microbes in African and Asian fauna there was less selection for tolerance.

## Actively acquired immune tolerance

Cellular immunologists sometimes protest against applying the word “tolerance” to describe asymptomatic viral infections, but that condition is the origin of the concept. Lifelong, asymptomatic replication and shedding of lymphocytic choriomeningitis virus (LCMV) in mice was revealed by Traub.^93^ When LCMV infection begins *in utero,* mice acquire the ability to tolerate viral replication throughout postnatal life without the brain disease and convulsions that follow adult‐onset infection. Traub did not use the word “tolerance” but noted the similarities with plant immunity to tobacco ring spot virus: “Immune plants are always carriers of virus, although they show no signs of disease.”

Traub's observations, together with Owen's finding that antibodies were not made against foreign erythrocyte alloantigens encountered *in utero* in dizygotic cattle twins,^94^ stimulated Burnet and Fenner^95^ to conceive that a system existed to inhibit antibodies that bind normal components of the body: “If, in embryonic life, expendable cells from a genetically different race are implanted and established, no antibody response should develop against the foreign antigen when the animal takes on an independent existence.”.

Medawar and colleagues experimentally demonstrated Burnet and Fenner's idea by showing that neonatal inoculation with allogeneic hematopoietic stem cells resulted in “actively acquired immunological tolerance” to foreign skin grafts that would normally have been swiftly rejected.^96^ In parallel, the concept of genetically encoded “pathogen tolerance” was developed by plant breeders who showed experimentally this was a beneficial trait to limit damaging immune responses against infectious microbes.^18^, ^19^

## Checkpoints for actively acquired immune tolerance

**Figure 4:**
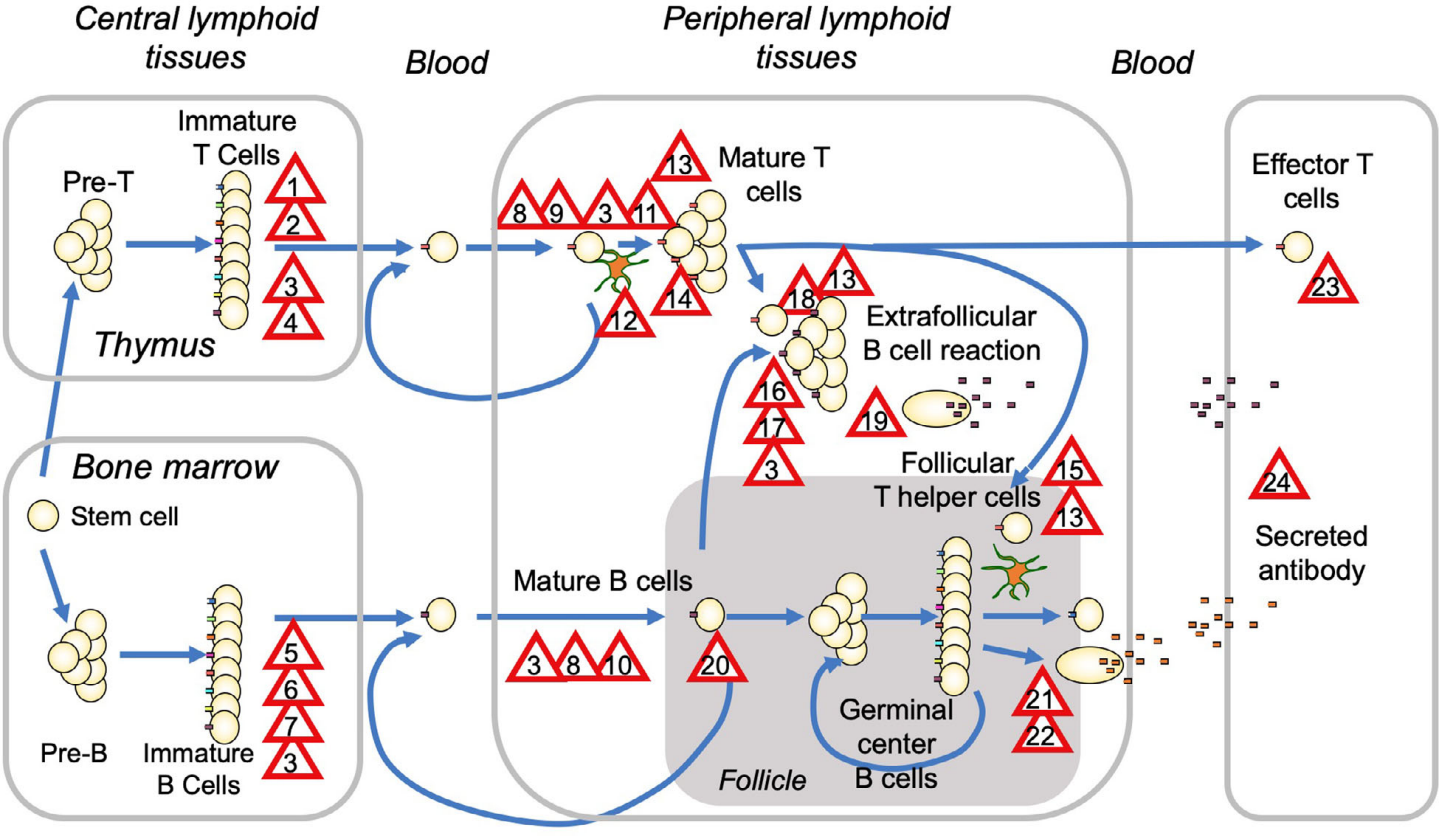
Sequential immune tolerance checkpoints balance antiviral immune responses with tolerance. Red triangles denote genetically separate checkpoints: (1, 2) two waves of thymic negative selection; (3) reversible anergy and inhibitory signaling by antigen receptor and inhibitory receptors; (4) FoxP3^+^ T‐regulatory cell differentiation; (5) reversible arrest of immature B‐cell maturation; (6) editing receptors by V(D)J recombination; (7) apoptosis of immature B cells; (8) apoptosis triggered by T‐cell receptor (TCR) or B‐cell receptor (BCR) induction of Bcl‐2 interacting mediator of cell death (BIM); (9 and 10) competition for survival factors, for example, interleukin (IL)‐2, IL‐7, IL‐15 and peptide/major histocompatibility complex for T cells or BAFF for B cells; (11) CD28, IL‐1, interferon costimulation of T‐cell clonal growth and inhibition by CTLA4, PD‐1 and other inhibitory receptors; (12) perforin‐ or FasL‐mediated elimination of infected or antigen‐bearing cells by activated T cells; (13) suppression of T and B cells by CTLA4^+^ Treg cells and TGF‐β; (14) FasL‐induced apoptosis of T cells; (15) repression of inducible T‐cell costimulator and T‐follicular helper cell differentiation by ROQUIN; (16) limiting provision of CD40L, IL‐2, IL‐4, IL‐6, IL‐21 for extrafollicular B‐cell growth and plasma cell differentiation; (17) repression of Toll‐like receptor‐induced B‐cell growth; (18) CD86–CD28–FasL checkpoint control of T‐cell help for B‐cell proliferation; (19) control of plasma cell formation by immunoglobulin M *versus* immunoglobulin G cytoplasmic tail and the BCR–ERK inhibitory pathway; (20) inhibition of B‐cell migration to follicles and recirculation; (21) competition for CD40L, IL‐4, IL‐21 from follicular helper T cells; (22) clonal redemption by selection for BCR mutations that remove self‐reactivity; (23) inhibition of effector T‐cell functions in tissues by PD‐1 ligands and dependence on major histocompatibility complex upregulation by interferons and (24) control of antibody extravasation and inflammation in tissues. Figure adapted from Goodnow 1996.^101^ ERK, extracellular signal‐regulated kinase; TGF‐β, transforming growth factor‐beta.

Contrary to Burnet and Fenner's idea that tolerance could only be acquired *in utero* by clonal deletion of cells with self‐reactive antibodies (before these antibodies could be tested for their utility in binding foreign microbes), immunological self‐tolerance is actively acquired throughout life by sequential checkpoints that balance tolerance with immunity.^97^, ^98^ A series of immune tolerance checkpoints (Figure 4) preserve the immune repertoire by tuning the activation, survival, proliferation and effector differentiation of B and T lymphocytes with self‐reactive receptors at each step in the formation of antibodies and effector T cells.^99^, ^100^, ^101^, ^102^

Tolerance checkpoints in B and T lymphocytes balance activating and inhibitory receptors and intracellular signals, switching an individual receptor between stimulating immunity and stimulating tolerance. This was first revealed by surface immunoglobulin antigen receptors on B cells where differences in the tempo and strength of stimulation, together with costimuli from other inhibitory (e.g. CD22, FAS, FCGR2B) or activating (e.g. CD21, CD40, IL‐4R) receptors, change the pattern of intracellular signals and genetic responses: either to trigger clonal proliferation and antibody secretion or to inhibit the maturation and survival of the B cell and actively establish tolerance.^103^, ^104^, ^105^, ^106^, ^107^, ^108^

**Figure 5:**
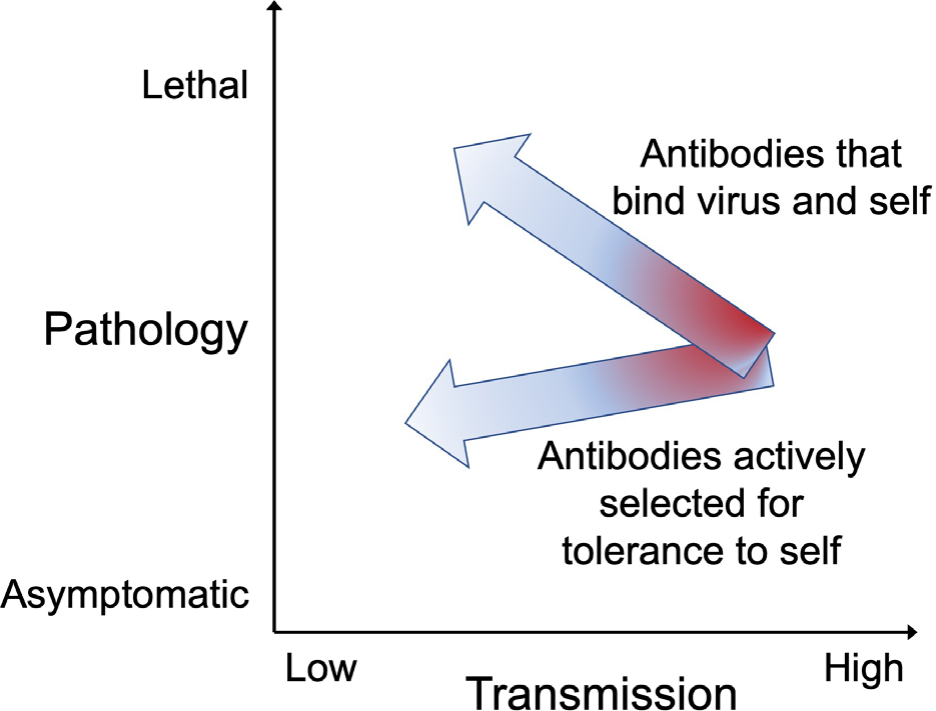
Transmission‐blocking antibodies can increase pathology if they also bind to self‐molecules, requiring multiple tolerance checkpoints to select for antibodies that are less self‐reactive and more virus specific.

Without self‐tolerance checkpoints, the production of neutralizing antibodies could paradoxically increase pathology during an infection (Figure 5). This is best illustrated by antibodies employing the *IGHV4‐34* gene in adult volunteers following vaccinia virus infection.^109^ Tracing the DNA sequence origins of these antibodies showed they started out as potentially damaging autoantibodies against self‐erythrocytes that also bound weakly to the virus. Prior to vaccination, the B cells bearing these antibodies would not have been clonally deleted but instead circulated through the body silenced against antibody secretion by the series of tolerance checkpoints.^110^ In response to binding vaccinia virus, the *IGHV4‐34* B cells were released from some checkpoints, proliferated and acquired somatic mutations in their antibody genes. Daughter cells were selected with antibody mutations that removed self‐reactivity and the risk of autoimmune hemolytic anemia, while other antibody mutations were selected that improved binding to the virus.^109^ Tracing this “clonal redemption” process in mice reveals how tolerant B cells in the spleen are reactivated by a sufficiently potent immunization and follow antibody mutation trajectories that cleanly remove self‐reactivity within 4–7 days, followed by targeted affinity maturation to fit unique features of the foreign immunogen.^111^, ^112^

The limits on tolerance during antiviral antibody responses are illustrated by chronic infection with hepatitis C virus. Broadly neutralizing antibodies against hepatitis C virus primarily use the *IGHV1‐69* gene, which encodes structural features that target a key site on the virus envelope E2 protein.^113^, ^114^, ^115^ However, these same *IGHV1‐69*‐encoded structural features confer polyreactivity with many other molecules, including frequent binding to the Fc domain of human immunoglobulin G as a rheumatoid factor autoantibody.^116^, ^117^ Indeed a subset of people with chronic hepatitis C virus develop *IGHV1‐69* rheumatoid factors that become extremely pathogenic causing mixed cryoglobulinemic vasculitis.^118^, ^119^ Because of the action of tolerance checkpoints, B cells bearing these *IGHV1‐69*‐encoded antibodies normally make only transient antibody responses and fail to accumulate as memory B cells.^120^

## Tolerance checkpoints in peripheral T cells

The concept of sequential tolerance checkpoints developed for B cells was subsequently extended to T cells, with the T‐cell receptor integrating tempo and strength of antigen stimulation with costimuli from inhibitory (CTLA‐4, PD‐1) and stimulatory (CD28, IL‐2R, IL‐1R, IFN) receptors.^121^, ^122^, ^123^ Chronic LCMV infection of adult mice illustrates this best. WhileTraub's state of chronic, asymptomatic LCMV infection was established by infection *in utero*, a similar state arises following infection of adult mice with a subclone of LCMV (clone 13) that was initially isolated from an *in utero* infected animal. In adult mice infected with LCMV clone 13, CD8 T cells bearing T‐cell receptors against viral peptides initially proliferate to form large clones but when these are unable to control chronic virus replication the T cells switch into a tolerant state of “exhaustion.” The tolerant state comes in part by expressing high levels of the inhibitory receptor, PD‐1,^124^ which is also essential for tolerance to self‐tissues.^123^

The burden of somatic mutations in cancer cells creates neoantigens that are not “self.” While immune responses initially retard growth of tumors bearing these neoantigens,^125^ these neoantigens become tolerated by the adaptive immune system and allow cancer spread. That tolerance can be overcome, and switched into an immune response, by antibody‐based drugs that block the inhibitory receptors responsible for key immune tolerance checkpoints: CTLA‐4^122^ and PD‐1.^123^ This use of checkpoint inhibitors has turned the concept of immune tolerance checkpoints to practical use, revolutionizing cancer therapy.

People whose CTLA4 checkpoint is blocked by antibody immunotherapy,^126^, ^127^, ^128^ or by germline mutations in the *CTLA4* gene,^129^, ^130^, ^131^ often but not always develop severe gastrointestinal inflammatory disease and autoimmune diseases. The variability between people in these adverse events highlights the crossover between tolerance of microbes, tolerance of self and cancer‐tolerance and the need to understand why it is more robust in some people than others.

## Pathological immune responses in COVID‐19 and SARS

Upon SARS‐CoV‐2 infection, many people exhibit mild or no clinical symptoms or radiographic evidence of lung disease despite actively shedding viral RNA and transmitting the infection to others who develop severe pneumonia.^4^, ^5^ This implies that viral replication in the airway epithelium is not highly pathogenic in its own right. In people with severe respiratory distress following SARS‐CoV‐2 infection there appears to be no discernable difference in virus RNA in respiratory secretions.^3^, ^5^ By contrast, there is clear laboratory evidence of a hyperinflammatory state in the blood resembling hemophagocytic lymphohistiocytosis with elevated C‐reactive protein, IL‐6 and D‐dimer.^132^, ^133^, ^134^, ^135^, ^136^, ^137^ These findings imply a failure to tolerate the virus, but which of the tolerance mechanisms discussed earlier are not functioning?

The incidence of severe COVID‐19 is much higher in older people, and the same was true for SARS. This is difficult to dissect because so many things change with age, including clonal hematopoietic stem cell mutations that exaggerate inflammatory responses.^138^ For SARS, comparison of old and young macaques infected for 4 days with SARS‐CoV found exaggerated lung inflammation and induction of numerous inflammatory messenger RNAs centered on nuclear factor kappa B‐induced genes in old animals.^139^ Despite comparable virus RNA, young animals made more interferon beta. Treatment of old animals with pegylated interferon alpha at the time of infection diminished virus‐associated inflammation. Thus, one explanation for increased disease severity in older people is their lung epithelial cells make less interferon to control virus replication, requiring a greater immune response through inflammatory pathways such as pyroptosis, IL‐1 and nuclear factor kappa B. That hypothesis is made much stronger by the recent finding of genetic defects or neutralizing autoantibodies that interfere with type 1 interferon signaling in a small but significant subset of people with severe COVID‐19.^140^, ^141^

Serum immunoglobulin G against SARS‐CoV appears on days 10–20 after symptom onset and may contribute to the decline in viral RNA in nasopharyngeal and fecal samples on days 15 and 20 after a peak on day 10.^142^ Because severe clinical worsening in a subset of patients occurred during this declining phase of virus production, this also points to an overexuberant immune response. Three independent studies found that people who subsequently developed severe or fatal SARS made higher titers of neutralizing antibodies on days 10–15 following onset of symptoms.^143^, ^144^, ^145^ A similar trend is emerging for higher antibody in people with more severe COVID‐19, but in both diseases the heightened antibody may simply be a marker of an exaggerated immune response and not necessarily a contributing factor.

Antibody responses can cause pathological reactions to virus replication, either through polyreactivity and self‐reactivity as discussed earlier or by the phenomenon of “antibody‐dependent enhancement.” The latter is an insurmountable barrier to vaccine control of a widespread coronavirus disease in cats, feline infectious peritonitis, which is made much worse because of antibody responses against the virus. In feline infectious peritonitis, even potent neutralizing monoclonal antibodies paradoxically enhance virus‐induced pathology because the antibody Fc segment enables antibody‐bound virus to enter cells bearing Fc receptors.^146^

It is unclear whether a similar mechanism contributes to severe SARS and COVID‐19 disease. Experimental evidence supporting this possibility has come from vaccination of macaques with a recombinant vaccinia virus expressing the SARS‐CoV spike protein.^147^ The vaccine elicited immunoglobulin G antibodies against SARS‐CoV that neutralized the virus in tissue culture, but instead of protecting the macaques from experimental infection, these antibodies caused severe lung disease characterized by a hyperinflammatory macrophage response. Earlier tissue culture experiments also raised the specter of antibody‐dependent enhancement of SARS. Immunoglobulin G from convalescent human sera or as mouse monoclonal antibodies promoted nonproductive virus replication in human macrophages and B cells in tissue culture, depending upon the ITAM‐bearing cytoplasmic tail of the activating Fc‐receptor, FcγR2a.^148^

## COVID‐19 and tolerance checkpoints: known unknowns

The current COVID‐19 crisis shines a beacon on the need to answer “known unknowns” about the mechanisms responsible for varying tolerance and immune response posed by every zoonotic leap from animals into humans. Several fundamental questions need to be answered:

- With respect to transmission, why are neutralizing antibody titers low and short‐lived following infection with COVID‐19, SARS and other coronaviruses? What titers and against which parts of the spike protein are needed to stop community transmission, and how can these be durably induced by a vaccine if the natural infection does not?
- On the pathology side, is severe COVID‐19 caused by inadequate (e.g. type 1 interferon) or by exaggerated (e.g. pyroptosis) innate immune responses? Or is it caused by inadequate or unchecked CD8 T‐cell responses, or by pathological antibody responses (polyreactive, self‐reactive or antibody‐dependent enhancement)? Are there particular immune tolerance checkpoints that fail in people with severe COVID‐19, and why is this more common as we get older?

Pinpointing and targeting the mechanisms responsible for varying pathology and inadequate antibody to stop transmission were out of reach in earlier coronavirus zoonoses. But this time around medical science is armed with the necessary genomic technologies and knowledge of immune response and tolerance checkpoint genes. It is time to deploy.

## Conflict of Interest

I have no conflicts of interest to declare.
